# Renal vein evaluation in venous excess ultrasound (VExUS): is it necessary?

**DOI:** 10.1093/ckj/sfaf205

**Published:** 2025-06-26

**Authors:** Masafumi Sakai, Keisuke Yoshida, Kana Shirai, Fumiya Kitano, Yugo Shibagaki, Masahiko Yazawa

**Affiliations:** Division of Nephrology and Hypertension, Department of Internal Medicine, St. Marianna University School of Medicine, Sugao, Miyamae-ku, Kawasaki, Kanagawa, Japan; Department of Internal Medicine, Yokohama General Hospital, Kurogane-cho, Aoba-ku, Yokohama, Kanagawa, Japan; Division of Nephrology, Department of Internal Medicine, Gyoutoku General Hospital, Hongyoutoku, Ichikawa, Chiba, Japan; Division of Nephrology and Hypertension, Department of Internal Medicine, St. Marianna University School of Medicine, Sugao, Miyamae-ku, Kawasaki, Kanagawa, Japan; Division of Nephrology and Hypertension, Department of Internal Medicine, St. Marianna University School of Medicine, Sugao, Miyamae-ku, Kawasaki, Kanagawa, Japan; Division of Nephrology and Hypertension, Department of Internal Medicine, St. Marianna University School of Medicine, Sugao, Miyamae-ku, Kawasaki, Kanagawa, Japan; Division of Nephrology and Hypertension, Department of Internal Medicine, St. Marianna University School of Medicine, Sugao, Miyamae-ku, Kawasaki, Kanagawa, Japan; Department of Internal Medicine, Yokohama General Hospital, Kurogane-cho, Aoba-ku, Yokohama, Kanagawa, Japan

To the Editor,

Since the venous excess ultrasound (VExUS) grading system was first proposed as a bedside tool for evaluating venous congestion using ultrasound [1], visualization of the renal vein has remained a technical challenge in the traditional VExUS (tVExUS) approach [2]. The clinical utility of a modified venous excess ultrasound (mVExUS) protocol—excluding renal vein evaluation—for the assessment of venous congestion has recently gained attention [2]. As VExUS becomes increasingly integrated into routine nephrology practice, particularly for guiding volume and congestion management, the question of whether renal vein assessment is essential warrants further discussion. The exclusion of this technically challenging component may enhance feasibility, but its diagnostic value remains a matter of debate [2]. This study investigated whether the mVExUS protocol, which omits renal vein assessment, could detect elevated right atrial pressure (RAP) as accurately as the tVExUS, using right heart catheterization (RHC) as the reference standard. Ninety-five patients undergoing RHC received VExUS examinations prior to the procedure. The area under the curve (AUC) for detecting elevated RAP (>12 mmHg) was 0.87 for tVExUS and 0.85 for mVExUS, indicating that both methods offer comparable diagnostic performance with high sensitivity and specificity [2].

We recently reported on the effects of a local educational program focused on point-of-care ultrasound (POCUS), including VExUS, and evaluated its clinical impact [3]. Prior to this program, VExUS had not been utilized at our institution. However, following a series of lectures and hands-on training sessions, the use of VExUS in cases of acute kidney injury increased significantly. Among the components of the VExUS grading system, the hepatic and portal veins were generally well visualized, whereas imaging of the renal vein remained technically challenging—being unsuccessful in nearly half of the cases [3]. Obtaining reliable Doppler waveforms of the renal interlobar veins is challenging due to the need for technical adjustments (e.g. flow velocity range and colour gain) and the requirement for patient breath-holding [3]. In particular, its utility may be limited in patients with chronic kidney disease and those with end-stage kidney disease undergoing haemodialysis, owing to potential alterations in renal perfusion in these populations [4, 5]. This underscores a practical limitation for clinicians. Therefore, the finding that mVExUS, which omits renal vein assessment, can evaluate venous congestion as effectively as tVExUS is of considerable clinical significance. In this regard, the demonstrated effectiveness of mVExUS supports its broader application in routine clinical practice.

On the other hand, the NephroPOCUS.com website (https://nephropocus.com/2025/03/27/hepatic-vein-doppler-and-ekg-are-there-any-workarounds/) has proposed a method for identifying the S and D waves in the hepatic vein by referencing renal vein and renal artery waveforms, thereby eliminating the need for electrocardiographic guidance. Additionally, it has been reported that significant tricuspid regurgitation can alter hepatic vein waveforms and that portal vein waveforms may be affected in patients with hypotensive cirrhosis complicated by gastrointestinal bleeding [6]. In such circumstances, reliance solely on hepatic and portal vein assessments may be insufficient to distinguish between moderate and severe venous congestion. Thus the ability to visualize the renal vein remains important and should be regarded as a necessary skill when performing VExUS. In fact, our investigation has shown that continuous on-the-job training can improve the implementation rate of renal vein visualization over time. These findings suggest that POCUS educational programs should place particular emphasis on developing the technical skills required for renal vein assessment [3].

However, it is also true that renal vein visualization remains challenging in many cases. Therefore, it is essential to recognize the limitations and appropriate clinical indications of mVExUS and to interpret its findings within that context. Accordingly, further research is warranted to identify specific patient cohorts in whom mVExUS may be particularly useful, as well as those for whom a full tVExUS assessment remains beneficial for the accurate evaluation of venous congestion.

## Data Availability

All clinical data were available from the electronic medical records at St. Marianna University Hospital. The data supporting the findings of this study are available from the corresponding author upon reasonable request.
